# Mechanism or Marker? Bridging Experimental and Epidemiological Evidence on Fatty Acids and Chronic Disease

**DOI:** 10.3390/nu18213500

**Published:** 2026-10-25

**Authors:** Cristina Sánchez-Quesada

**Affiliations:** 1 Preventive Medicine and Public Health Division, Department of Health Sciences, Faculty of Health Sciences, University of Jaén, 23071 Jaén, Spain; csquesad@ujaen.com; Tel.: +34-953-211-754; 2 University Institute of Research on Olive and Olive Oils (INUO), University of Jaén, 23071 Jaén, Spain; 3 Agrifood Campus of International Excellence (ceiA3), 14071 Córdoba, Spain

## 1. Introduction

Chronic non-communicable diseases remain the leading source of morbidity and mortality in ageing populations, and diet is one of the few modifiable determinants shared by most of them. Metabolic syndrome, which affects an estimated 20–25% of the adult population worldwide, illustrates this overlap: it clusters abdominal obesity, dyslipidaemia, hypertension, and hyperglycaemia, and it increases the risk of type 2 diabetes, cardiovascular disease, and all-cause mortality [1]. Atrial fibrillation, the most prevalent sustained cardiac arrhythmia, now affects roughly 59 million people and carries an approximately five-fold increase in stroke risk [2,3,4]. Despite their different clinical presentations, both conditions are sustained by low-grade inflammation, oxidative stress, and tissue remodelling, which are the processes through which dietary lipids are thought to exert their effects.

For decades, nutritional guidance has focused on the quantity of total dietary fat. This view has gradually been replaced by one centred on fat quality, that is, the fatty acid composition of the diet. Saturated fatty acids have been linked to oxidative stress, endoplasmic reticulum stress, and inflammation, whereas monounsaturated (MUFAs) and polyunsaturated fatty acids (PUFAs) have been associated with more favourable metabolic profiles [5,6]. Within the PUFA family, long-chain omega-3 fatty acids, namely eicosapentaenoic acid (EPA) and docosahexaenoic acid (DHA), are precursors of specialised pro-resolving mediators such as resolvins, maresins, and protectins, whereas omega-6 fatty acids give rise mainly to pro-inflammatory eicosanoids [3]. Because the endogenous conversion of the plant-derived precursor α-linolenic acid (ALA) into EPA and DHA is limited, the dietary source of these fatty acids is a relevant determinant of their availability [3,7].

However, translating this biological rationale into clinical and public health recommendations has proved difficult. Evidence from supplementation trials is inconsistent, and pharmacological doses of marine omega-3 fatty acids have been associated with a higher incidence of atrial fibrillation, a signal not observed with habitual dietary intakes [7]. Moreover, the effects of fatty acids appear to depend on the tissue studied, the life stage, the dose, and the food or pharmacological source, and some relationships may be non-linear [5,7].

The recent collection published in *Nutrients* on the role of fatty acids in chronic disease development reflects this complexity. It brings together evidence from experimental models of adipose tissue dysfunction [8], from human milk composition in maternal obesity [9], from dietary MUFA intake and metabolic syndrome in a national survey [10], from omega-3 intake and biomarkers of atrial fibrillation pathways in a Mediterranean cohort [11], and from pro-resolving lipid mediators in autoimmune thyroiditis [12]. Taken together, these studies span the life course, from lactation to older age, and the translational spectrum, from murine models to population-based observation.

The aim of this work is to critically review this evidence, to identify the novel contributions of these studies to the understanding of fatty acids in chronic disease, and to discuss the methodological limitations that currently constrain their interpretation and their translation into preventive practice.

## 2. Biochemical and Pathophysiological Bases

Fatty acids are classified as saturated, monounsaturated (MUFA), and polyunsaturated (*n*-3 and *n*-6). The conversion of α-linolenic acid (ALA) into eicosapentaenoic (EPA) and docosahexaenoic acid (DHA) is inefficient, so long-chain *n*-3 intake depends largely on marine sources [11,12]. EPA and DHA give rise to specialised pro-resolving mediators (resolvins, maresins, protectins), whereas *n*-6 arachidonic acid (AA) yields mainly pro-inflammatory eicosanoids [12,13]. The *n*-3/*n*-6 balance is therefore a determinant of inflammatory tone [9,12].

## 3. Early-Life Programming

In a small study of lactating women (*n* = 20), maternal overweight/obesity was associated with milk richer in fat and total *n*-3 and *n*-6, yet with similar foremilk DHA, lower hindmilk DHA, and a higher AA/DHA ratio [9]. Plasma *n*-3 and *n*-6 were roughly halved [9]. Reduced peroxisomal conversion of ALA to DHA under hyperinsulinaemia is proposed as a mechanism, but this remains a hypothesis, and the sample is too small to support supplementation recommendations [9].

## 4. Obesity and Adipose Tissue

In diet-induced obese mice, replacing lard with fish oil for 12 weeks improved autophagic flux (higher LC3-II/LC3-I, lower p62), reduced lipid peroxidation in visceral fat, and reduced subcutaneous adipocyte size [8]. Lard, coconut, and olive oil did not restore autophagic flux, although olive oil improved antioxidant status [8]. The effects were depot-specific and body-weight differences were not significant [8]. Extrapolation to humans is limited.

## 5. Cardiometabolic Disease

In 14,817 Korean adults, higher MUFA intake was associated with lower odds of metabolic syndrome only in middle-aged men (OR 0.52) and women (OR 0.66), mainly through triglycerides [5]. Findings were cross-sectional and based on a single 24 h recall [10]. In the PREDIMED-Plus subsample (*n* = 510), habitual total omega-3 intake (median 2.0 g/day) was not associated with five atrial fibrillation-related biomarkers over five years [11]. Isolated signals (marine *n*-3 with 3-nitrotyrosine, a non-linear association of non-marine *n*-3 with PICP) are best read as hypothesis-generating [11]. Habitual dietary intakes lie well below the supplemental doses linked to higher atrial fibrillation risk [11].

## 6. Chronic Inflammation and Autoimmunity

In 33 women with Hashimoto’s thyroiditis, EPA and DHA concentrations were not related to inflammatory or thyroid parameters, but several derived mediators (resolvin E1, protectin DX, 18-HEPE) correlated positively with C-reactive protein, and maresin 1 with free thyroxine [12]. The authors interpret this as a compensatory pro-resolving response, but the design cannot distinguish compensation from a mere marker of inflammation [12].

## 7. What Is New

Three contributions stand out: longitudinal biomarker trajectories instead of single measurements [11]; non-linear, source-dependent associations [8,11]; and a shift from total fatty acids towards lipid mediators and tissue-specific responses [8,12]. Taken together, the evidence supports fat quality as relevant to chronic disease, but not as a uniform or dose-independent effect [8,9,10,11,12].

## 8. Limitations of the Evidence and Sources of Bias

The studies reviewed share methodological constraints that limit causal interpretation and, above all, the translation of their findings into preventive recommendations.

Study design: The Korean analysis relates MUFA intake to the prevalence of metabolic syndrome at a single time point [10], and the milk study compares maternal plasma and milk composition at 7–9 weeks postpartum [9]; reverse causation cannot be excluded in either. The same applies to the Hashimoto’s thyroiditis cohort, where positive correlations between pro-resolving mediators and C-reactive protein are compatible with a compensatory response, but equally with a marker of inflammatory burden [12]. Even the longitudinal PREDIMED-Plus analysis is observational with respect to omega-3 intake, since the exposure was not randomised [11].

Exposure assessment: Dietary intake was captured with a single 24 h recall in the Korean survey, which cannot represent habitual intake [10]; with a 143-item food-frequency questionnaire in PREDIMED-Plus, where measurement error is expected to attenuate associations towards the null [11]; and with a three-day food diary in the Hashimoto’s cohort, whose habitual EPA and DHA intakes were very low [12]. The milk study does not report dietary intake, so differences in plasma and milk fatty acids cannot be separated into dietary and metabolic components [9]. The Korean study could not distinguish plant-derived from animal-derived MUFAs, which may have different health effects [10].

Sample size and multiplicity: The milk study analysed 20 women [9] and the Hashimoto’s study 33 [12]. In the murine study, most molecular analyses were performed on six animals per group [8]. Conversely, the PREDIMED-Plus authors acknowledge that 510 participants may be underpowered for small effects, and that chance in the context of multiple testing is a plausible explanation for the cross-sectional association between marine omega-3 and 3-nitrotyrosine [11]. The Hashimoto’s study tested numerous correlations, and no correction for multiple comparisons is reported [12].

Outcome validity: The circulating markers measured in PREDIMED-Plus are mechanistic surrogates rather than validated intermediate endpoints for atrial fibrillation [11]. Likewise, autophagy and lipid peroxidation markers in murine adipose tissue are not clinical outcomes, and glucose tolerance and insulin were not measured, so insulin resistance could not be confirmed [8].

Confounding and intervention composition: Residual confounding by overall Mediterranean diet adherence is difficult to separate from omega-3 intake despite adjustment [11]. In the murine study, the fish oil diet was based on cod liver oil, which also contained other fatty acids, such as arachidonic and paullinic acid, in non-negligible amounts [8]; its effects therefore cannot be attributed to EPA and DHA alone.

Generalisability and source of exposure: Evidence comes from male mice [8], older Spanish adults with metabolic syndrome [11], a nationally representative Korean sample [10], a small and ethnically mixed group of lactating women [9], and women with Hashimoto’s thyroiditis already treated with levothyroxine [12]. Habitual dietary omega-3 intakes also differ substantially from the pharmacological doses tested in trials, in which higher atrial fibrillation incidence has been reported [14,15]; findings from one source cannot be transferred to the other [11].

Overall, heterogeneity in populations, exposures, outcomes, and designs precludes quantitative synthesis. These studies are best regarded as hypothesis-generating and replication in larger, prospective, and, where feasible, interventional designs is required.

## 9. Conclusions

Impact of chronic diseases: Metabolic syndrome (affecting 20–25% of adults globally) and atrial fibrillation share underlying mechanisms such as low-grade inflammation, oxidative stress, and tissue remodelling, which can be influenced by dietary lipids.

Shift to Fat quality: Nutritional guidance has shifted from total fat intake to fat quality, highlighting that saturated fats promote inflammation and oxidative stress, whereas MUFAs and PUFAs are associated with healthier metabolic outcomes.

Role of omega-3 vs. omega-6: Long-chain omega-3 fatty acids (EPA/DHA) act as precursors to anti-inflammatory mediators, while omega-6 fatty acids mostly generate pro-inflammatory compounds. Direct dietary intake of EPA/DHA is essential due to limited internal conversion from ALA.

Clinical translation challenges: High pharmacological doses of omega-3 supplements have been linked to an increased incidence of atrial fibrillation (not seen in habitual dietary intake), showing that lipid effects vary based on dose, source, tissue type, and life stage.
